# How to diagnose heart failure with preserved ejection fraction: the value of invasive stress testing

**DOI:** 10.1007/s12471-016-0811-0

**Published:** 2016-02-25

**Authors:** A. E. Huis in ’t Veld, F. S. de Man, A. C. van Rossum, M. L. Handoko

**Affiliations:** Department of Pulmonology, Institute for Cardiovascular Research (ICaR-VU), VU University Medical Centre (VUmc), Amsterdam, The Netherlands; Department of Cardiology, ICaR-VU, VUmc, Amsterdam, The Netherlands

**Keywords:** Heart failure, Diastole, Diagnosis, Echocardiography, Exercise test, Swan-Ganz catheterisation

## Abstract

Heart failure with preserved ejection fraction (HFpEF) is a growing healthcare burden worldwide and its prevalence is increasing. Diagnosing HFpEF is challenging and relies upon the presence of symptoms and/or signs of heart failure, preserved left ventricular systolic function, and evidence of diastolic dysfunction. Current diagnostic algorithms mainly rely on echocardiography (E/e’) and biomarkers (NT-proBNP). However, only a minority of patients with HFpEF are identified, and especially HFpEF patients at an early stage of the disease are easily missed. We propose to incorporate invasive stress testing, by means of right heart catheterisation at rest and during exercise, and accurate assessment of right ventricular function, by means of cardiac magnetic resonance imaging. These additions to the current diagnostic work-up will improve diagnostic sensitivity and accurate staging of HFpEF patients.

## Heart failure with preserved ejection fraction

Heart failure with preserved ejection fraction (HFpEF, previously described as diastolic heart failure) is a clinical syndrome characterised by evidence of symptoms and/or signs of heart failure, left ventricular (LV) diastolic dysfunction, and -by definition- a preserved LV systolic function [1]. Although symptoms and signs of HFpEF are non-specific, individuals almost always present with dyspnoea on exertion and impaired exercise tolerance. While most clinical signs and symptoms in heart failure with preserved and reduced LV function are quite similar, individuals with HFpEF are more often older, predominantly female and are more likely to present with comorbidities, such as hypertension, obesity, diabetes, atrial fibrillation and renal disease [2, 3]. The exact pathophysiological mechanisms behind HFpEF are not fully understood, but likely to be multifactorial. LV diastolic dysfunction is the cornerstone process, but other factors may contribute to heart failure in these patients, including altered ventricular-arterial coupling, chronotropic incompetence, coronary artery disease, coronary microvascular rarefaction, fibrosis and even subtle systolic dysfunction [4–7]. In addition, non-cardiac comorbidities such as pulmonary disease, anaemia, renal dysfunction or obesity may, at least in part, be responsible for the symptoms of heart failure.

HFpEF currently accounts for approximately 50 % of new heart failure cases and its prevalence relative to heart failure with reduced ejection fraction (HFrEF) is increasing [8, 9]. Furthermore, unlike previously thought, morbidity and survival rates among patients with HFpEF are as ominous as for their counterparts with reduced ejection fraction [2]. Despite these worrisome trends, no significant improvements in therapeutic strategies for HFpEF have been established [9]. Part of the explanation as to why results of therapeutic trials focusing on HFpEF have been disappointing thus far might be related to large differences in the underlying pathophysiological profile in these patients, which may have led to the inclusion of a heterogenic group of patients at different stages of the disease. Careful inclusion of HFpEF patients according to their stage of the disease, rather than considering HFpEF patients as one homogeneous group, might help to overcome this problem.

## HFpEF: a diagnostic challenge

Although HFpEF poses a significant burden on healthcare systems worldwide, many questions regarding the best diagnostic approach remain unanswered. Diagnosing HFpEF is often a clinical challenge and this holds especially true for outpatients at an early stage of the disease without overt signs of heart failure [10, 11]. At that point the diagnosis is easily missed, as a normal ejection fraction and no evident signs of fluid retention may shift attention towards other causes of dyspnoea, such as pulmonary disease, obesity or even deconditioning. Additionally, the fact that specific treatment for this disease is lacking, that symptoms are often nonspecific, and that current non-invasive markers of diastolic dysfunction especially lack sufficient sensitivity, may enhance the tendency to under-diagnose this disease even more [11–13]. In this review, we will discuss current diagnostic algorithms, and argue for a wider use of invasive stress testing.

## Current diagnostic algorithms

Up to now, four guidelines reporting on diagnosing HFpEF have been published [1, 13–15]. We will mainly discuss the guideline by Paulus et al. [1] as it is the most recent and most often cited.

**Fig. 1:**
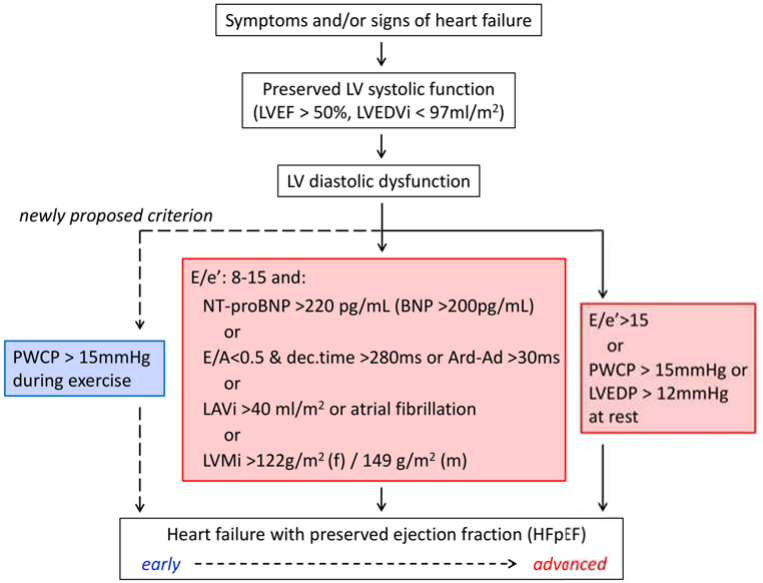
How to diagnose HFpEF. We propose ‘elevated PCWP during exercise’ as a new criterion for (early) HFpEF. *LVEF* left ventricular ejection fraction, *LVEDVi* indexed left ventricular end-diastolic volume, *PWCP* pulmonary capillary wedge pressure, *dec.time* deceleration time, *LAVi* indexed left atrial volume, *LVMi* indexed left ventricular mass, *LVEDP* left ventricular end-diastolic pressure

Following this guideline, patients have to fulfil three criteria: signs and/or symptoms of heart failure, no impaired systolic LV function (LV ejection fraction > 50 % and indexed LV end-diastolic volume < 97 ml/m²) and evidence of LV diastolic dysfunction. Regarding evidence of diastolic dysfunction, the guideline by Paulus et al. states that either invasive haemodynamic measurements (pulmonary capillary wedge pressure (PCWP) > 15 mmHg or LV end-diastolic pressure > 12 mmHg at rest) or tissue Doppler measurements (E/e’ >15) provide sufficient stand-alone evidence of diastolic dysfunction. E/e’ in the range of 8–15 or high NT-pro B-type natriuretic peptide (NT-proBNP) levels (> 220 pg/ml; or BNP > 200 pg/ml) need to be accompanied by at least one additional sign of diastolic dysfunction, including a low E/A ratio combined with a high deceleration time, pulmonary venous flow patterns suggestive of diastolic dysfunction, high indexed left atrial volume, the presence of atrial fibrillation, and/or LV hypertrophy (Fig. 1).

So far, validation of the above-mentioned guidelines in large populations is limited, especially in patients at an early stage of HFpEF, where resting filling pressures are still normal. Next, we will discuss the usefulness and the most important drawbacks of the proposed non-invasive measurements for diastolic dysfunction.

## Echocardiography: its role in diagnosing HFpEF

Echocardiography has a pivotal role in the diagnostic process of HFpEF and is generally considered one of the most useful tests in this setting. It is readily available, provides information on general cardiac anatomy and it allows the estimation of filling pressures combined with good spatial and temporal resolution. Provided diastolic dysfunction is the hallmark pathophysiological process in HFpEF, it is not surprising that echocardiography in HFpEF mainly focuses on markers of diastolic dysfunction. However, as echocardiographic parameters of diastolic dysfunction are influenced by heart rate and loading conditions, the main question is whether echocardiography, and Doppler parameters in particular, are suitable for assessing diastolic dysfunction.

Besides E/e’, which will be discussed below, echocardiographic indices of diastolic dysfunction include E/A ratio, deceleration time of E, pulmonary venous flow assessment, left atrial (LA) volumes and LV mass [1]. Their usefulness in the diagnostic approach of patients suspected of HFpEF is narrowed to patients with an inconclusive E/e’ (between 8–15), as described previously [12]. E/A ratio as assessed by pulsed wave Doppler represents mitral valve filling velocities and it is directly dependent on the pressure gradient between the left atrium and left ventricle (e.g. atrioventricular pressure gradient). As a consequence, it is sensitive to both LA pressure and ventricular filling properties. However, due to the presence of pseudo normalisation and its preload dependence, it is no longer advised to use it as stand-alone evidence for diastolic dysfunction. The validity and predictive value of the remaining blood flow Doppler derived markers of diastolic dysfunction have been debated on by past authors and therefore the physiological principles and measuring techniques fall beyond the scope of this review [16–19].

The key echocardiographic measurement in assessing diastolic dysfunction is E/e’. E represents peak velocity of transmitral flow in early diastole, as assessed by pulsed wave Doppler, whereas e’ represents either the early diastolic septal or lateral lengthening peak velocity of the mitral annulus, measured with tissue Doppler. As such, E is considered a reflection of the maximum pressure differences between the left atrium and left ventricle, and thus is mainly dependent on both ventricular relaxation and left atrial pressures. E’ is a reflection of the amount of blood entering the ventricle and mainly related to ventricular relaxation/LV filling pressures. E/e’ is thought to be a reflection of left atrial pressures and thus of left ventricular end-diastolic pressure. E/e’ is generally assumed to be less sensitive to preload than other echocardiographic indices of diastolic dysfunction and therefore yields more accurate estimations of filling pressures.

To date, an elevated E/e’ (reflecting filling pressures > 15 mmHg) is incorporated in guidelines as sufficient evidence of diastolic dysfunction [1]. Evidence supporting this approach comes from a study conducted by Ommen et al. [20]. Although in that study the correlation coefficient of E/e’ and mean LV filling pressures was only 0.47, all patients with a E/e’ >15 had high invasively assessed filling pressures, suggesting an excellent specificity of E/e’. Furthermore, other authors stated good correlations of E/e’ and invasively assessed LV filling pressures [21–23]. In contrast, it has been found that the correlation between E/e’ and PCWP may be worse than previously reported, albeit in either acutely decompensated patients with systolic dysfunction (LVEF < 30 %), [24] or symptomatic patients with hypertrophic cardiomyopathy [25]. Also, it has been shown that changes in filling pressures over time in individual patients do not correlate with changes in E/e’, hampering the use of E/e’ as a tool to monitor individual patients during their disease course or monitor their response to therapy [18].

Although signs of diastolic dysfunction in terms of an elevated E/e’ >15 seem sufficient to detect elevated LV pressures in the initial assessment of patients suspected of HFpEF, filling pressures are variable over time, depending on volume status. Moreover, filling pressures might not be elevated in an early stage of the disease, as demonstrated by a study by Penicka et al. [11]. They found that in 30 % of stable outpatients with unexplained dyspnoea, invasively proven to have HFpEF, E/e’ indices were normal. Furthermore, only 25 % of HFpEF patients fulfilled the current definition of HFpEF and 20–40 % of controls had borderline E/e’ values, suggesting both low specificity and sensitivity for E/e’, even in combination with additional echo markers of diastolic dysfunction. Santos et al. recently reported that E/e’ could not accurately estimate PCWP, as demonstrated by Bland-Altman analysis and they showed that E/e’ did not accurately reflect changes in PCWP during alterations in loading conditions [26].

Taking into account the last-mentioned studies that have called the utility of E/e’ into question, [11, 26] it seems that E/e’ is not sensitive enough to detect HFpEF in outpatients with unexplained dyspnoea in an early stage of the disease, when impairments in diastole are less prominent. The absence of an elevated E/e’ therefore does not rule out the presence of diastolic dysfunction.

## Biomarkers: value of BNP or NT-proBNP in diagnostic process

BNP is predominately produced by ventricular myocardium and its release is stimulated by ventricular wall stress. Consequently, elevated plasma levels of BNP (or its biologically inactive form, NT-proBNP) directly reflect myocardial stretch and are an indirect measure of elevated filling pressures. High BNP or NT-proBNP levels are proven to be correlated with high filling pressures and severity of diastolic dysfunction, [27] and are a strong predictor of outcome [28]. Despite levels being lower in patients with HFpEF than in HFrEF, NT-proBNP is generally considered to be of value in the routine diagnostic work-up in patients with preserved ejection fractions [1]. However, NT-proBNP levels are also influenced by tachycardia, atrial fibrillation, myocardial ischaemia, obesity or renal dysfunction, yielding low specificity for this marker [29]. In addition, it is known that patients with near-normal or mildly elevated filling pressures can present with normal NT-proBNP levels [28, 30], and NT-proBNP also fails to reflect elevated filling pressures in patients who merely present with elevated filling pressures during exercise [11]. As such, although in contrast with the latest guideline, [1] we do not recommend using low NT-proBNP measures to exclude HFpEF, nor do we propagate elevated NT-proBNP levels as isolated evidence of diastolic dysfunction. The preferred approach is always to combine levels of NT-proBNP with additional measures of diastolic dysfunction.

## Diagnosing HFpEF: the value of invasive stress testing

In a significant proportion of patients with high clinical suspicion of HFpEF, previously described non-invasive measures are not sufficient to account for the presence of significant LV diastolic dysfunction. Importantly, the absence of elevated filling pressures at rest does not preclude patients from having profound haemodynamic impairments during exercise. Furthermore, filling pressures may be variable over time, depending on volume status and physical activity. As such, the assessment of elevated filling pressures non-invasively is far from an easy task.

## Diastolic stress testing: why and how

Exercise testing has been shown to enhance the diagnosis of HFpEF in patients with no overt signs of volume overload and normal filling pressures at rest and it holds the most promise for novel diagnostic strategies [31].

**Fig. 2:**
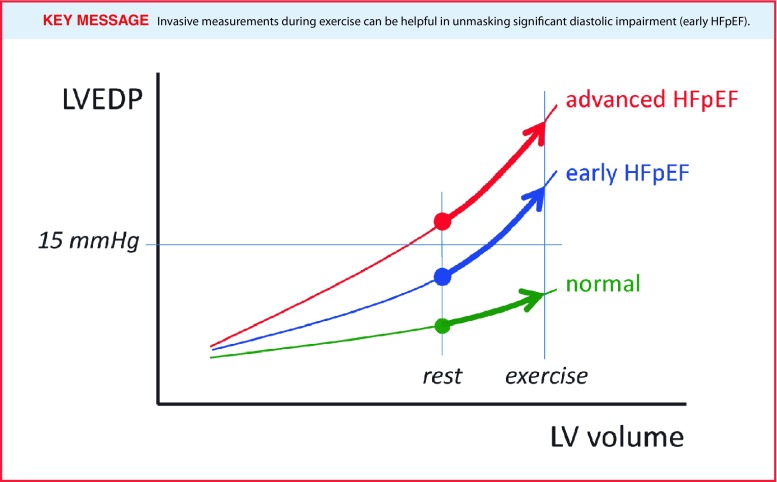
At rest, left ventricular filling pressures might be normal (LVEDP or PCWP < 15 mmHg), even though the left ventricular end-diastolic pressure-volume relationship is already disturbed/steepened. Invasive measurements during exercise can be helpful in unmasking significant diastolic impairment (early HFpEF)

In healthy subjects, exercise causes an increase in stroke volume, established by an increase in end-diastolic volume combined with a reduction in end-systolic volumes. As the heart rate increases as well, the left ventricle has less time to fill, whereas at the same time an elevated venous return causes an increase in volume loading. In healthy patients, a high diastolic reserve (the ability to increase myocardial relaxation under stress) prevents LV filling pressures from rising. In HFpEF, this normal diastolic reserve capacity is exhausted during exercise and LV filling pressures will increase (Fig. 2; [31, 32]). Additionally, a blunted increase in contractility during exercise has also been shown to play a role [33].

Up to now, various studies have reported on the use of exercise echocardiography (stress echocardiography) as a potential diagnostic tool [34–37]. Burgess et al. demonstrated that in unselected patients undergoing left heart catheterisation, E/e’ during exercise correlates with invasively measured LV filling pressures and that an elevated E/e’ at exercise can help to identify patients with high filling pressures during exercise [35]. In contrast, Maeder et al. reported that peak exercise E/e’ did not significantly differ between HFpEF patients and controls, and that it did not correlate with right heart catheterisation derived PCWP during exercise [12]. On the other hand, exercise echocardiography may help to identify patients at highest risk of cardiac hospitalisation or death, since Donal et al. recently demonstrated that estimated pulmonary artery pressures by tricuspid regurgitation maximal velocity during exercise yield prognostic information in HFpEF patients [34].

One of the first studies addressing markedly elevated filling pressures during moderate exercise in patients with normal resting haemodynamics was by Borlaug et al. [31]. In that study, it became apparent that more than 50 % of patients with dyspnoea on exertion showed increased LV filling pressures merely during exercise. Results from this study suggest that measuring PCWP during a right heart catheterisation can help unmask the presence of diastolic dysfunction in patients with unexplained dyspnoea at an early stage of the disease. Corroborating these findings, Andersen et al. identified a significant increase in PCWP with exercise in 26 patients with either high filling pressures at rest, or merely with exercise [38]. Interestingly, they directly compared haemodynamic responses to exercise with acute volume loading and found that the former was the more sensitive manoeuvre to identify patients with exercised-induced HFpEF. The utility of invasive exercise testing is even more enhanced by the recent finding that an excessive rise in PCWP with exercise (‘early’ HFpEF) is associated with increased mortality rates [39].

Despite these studies providing adequate evidence of the utility of exercise measurements, it is currently not included in the guidelines as a standard approach [1]. As the utility of echocardiographic parameters during exercise measures has been challenged and clear standardisation is lacking, [12] the use of invasively measured LV filling pressures during exercise is preferred and yields more robust information on haemodynamic derangements. Assessing filling pressures during right heart catheterisation is the favoured approach as it allows the simultaneous invasive assessment of pulmonary artery pressures, which can be used to further stratify patients. Furthermore, despite its invasive nature, right heart catheterisation is considered safe when performed at expert centres: morbidity and mortality rates are 1.1 and 0.055 % respectively [40].

Taken together, we therefore propose to use invasive exercise testing to identify patients with HFpEF, when non-invasive markers are inconclusive, no overt signs of fluid overload are present and clinical suspicion persists.

## Additional role for CMR

Over the last decade, attention has been given to the potential utility of cardiac magnetic resonance imaging (CMR) and it is among the most promising non-invasive modalities. It provides excellent spatial resolution, and allows the assessment of global and regional cardiac anatomy. It is considered the clinical gold standard for measuring LV volumes and function, including LV hypertrophy. Furthermore, left atrial volume and function, blood flow velocities, as well as information on myocardial tissue characteristics such as T1-mapping to quantify the degree of diffuse myocardial fibrosis, can be obtained [41–44].

In addition, CMR is also considered the gold standard for the assessment of the right ventricle [45]. Patients with HFpEF are at risk of developing pulmonary hypertension and thus right heart failure, as the right ventricle seems especially sensitive to alterations in afterload [46]. Therefore, CMR might help to risk stratify HFpEF patients, especially when echocardiography yields inconclusive results.

## Staging of HFPEF and the potential therapeutic consequences

Up to now, almost all HFpEF trials yielded either negative or inconclusive results. Advanced phenomapping techniques were able to demonstrate that HFpEF is a heterogeneous disorder (three phenotypes were identified with distinct survival), [47] for which a one-size-fits-all approach is probably not the solution. This knowledge should be implemented in future diagnostic strategies for HFpEF. Evidence supporting this approach is limited, but can be derived from recent HFpEF trials. For instance, targeting HFpEF with the phosphodiesterase-5 inhibitor sildenafil had no effect on outcome in a large multicentre trial, [48] whereas a small study conducted in a specific HFpEF population with advanced pulmonary vascular disease and right heart failure yielded beneficial results [49].

## Conclusion/recommendations

**Fig. 3:**
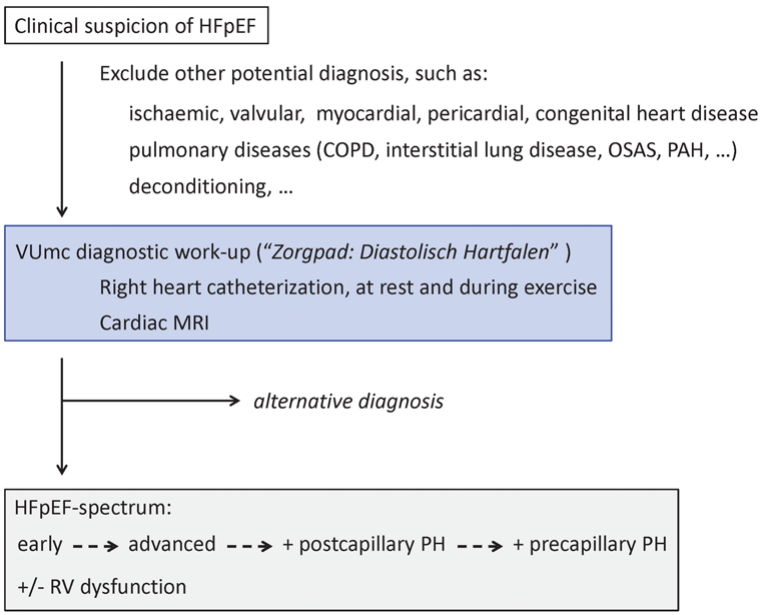
VUmc diagnostic work-up for HFpEF (‘Zorgpad: Diastolisch Hartfalen’). Referred patients with a clinical suspicion of HFpEF will undergo right heart catheterisation at rest and during exercise, as well as CMR. Either an alternative diagnosis is found, or HFpEF is confirmed and staged according to findings as: early HFpEF, advanced HFpEF, with or without post- or combined post- and pre-capillary pulmonary hypertension (PH), and with or without right ventricular (RV) dysfunction. *OSAS* obstructive sleep apnoea syndrome, *PAH* pulmonary arterial hypertension

The current diagnostic approach to HFpEF only identifies a minority of patients with HFpEF. We propose to incorporate invasive stress testing in the diagnostic work-up, by means of right heart catheterisation at rest and during exercise, and to include accurate assessment of RV function, by means of CMR. These additional measurements will not only help to identify patients with early HFpEF, but will also stage patients with advanced HFpEF accordingly, namely advanced HFpEF with and without pulmonary hypertension, and with or without right ventricular dysfunction. In early 2016, the VU University Medical Center will start with a specific diagnostic care pathway for HFpEF (‘Zorgpad: Diastolisch Hartfalen’), in which the proposals from this review will be incorporated (Fig. 3). Future studies are necessary to evaluate whether diagnostic refinements in HFpEF ultimately result in therapeutic benefit for the patient.
